# Bioactive Compounds and In Vitro Antioxidant Capacity of Cambuci and Uvaia: An Extensive Description of Little-Known Fruits from the Myrtaceae Family with High Consumption Potential

**DOI:** 10.3390/foods11172612

**Published:** 2022-08-29

**Authors:** Isabela Barroso Taver, Poliana Cristina Spricigo, Horst Bremer Neto, Severino Matias de Alencar, Adna Prado Massarioli, Angelo Pedro Jacomino

**Affiliations:** Departamento de Produção Vegetal, Escola Superior de Agricultura “Luiz de Queiroz”, Universidade de São Paulo, Piracicaba 13418-900, SP, Brazil

**Keywords:** *Campomanesia phaea* O. Berg Landrum, *Eugenia pyriformis* Cambess, ABTS, ORAC, HOCl

## Abstract

Cambuci (*Campomanesia phaea* O. Berg Landrum) and uvaia (*Eugenia pyriformis* Cambess), both native Atlantic Rainforest fruits, are noteworthy for being rich in bioactive compounds and their significant antioxidant capacity. Despite the numerous known edible fruits in the world, consumption by humans is most often restricted to a few dozen of them. Such behavior occurs, among other reasons, due to the lack of knowledge about fruits not yet commercialized on a large scale. This study quantified the bioactive compound content (total phenolic compounds and ascorbic acid in cambucis and uvaias; proanthocyanidins in cambucis, and total carotenoid profile and individual carotenoids for grapes) and antioxidant capacity of the edible parts (peel and pulp) of cambuci and uvaia accessions, using three methods (ABTS•+, ROO• radical scavenging and HOCl elimination). Cambuci contained higher phenolic compound levels and displayed higher antioxidant capacity determined by the ABTS•+ and ROO• radical scavenging methods than uvaia (139 and 119 mg 100 g^−1^ of GAE, 10.5 and 7.73 μmol g^−1^ of TE; 9.17 and 5.92 μmol g^−1^ of TE, respectively). Vitamin C content and the antioxidant capacity determined by the HOCl elimination method were about 1.5- and 6-fold higher in uvaia compared to cambuci, with the latter being a first-time report for uvaia. Both fruits contained higher levels of bioactive compounds and antioxidant capacity than other commonly consumed fruits.

## 1. Introduction

The United Nations Food and Agriculture Organization (FAO) declared 2021 as the International Year of Fruits and Vegetables, seeking to stimulate vegetable consumption and raise awareness of their nutritional benefits and on human health issues [1]. Plant-based diets are associated with a reduced risk of non-communicable chronic diseases (NCCDs), mainly as the intake of plant-based food items supplements human diets with bioactive compounds and antioxidants, which protect the body and promote health benefits [2].

Brazil is the third largest fruit producer in the world, ranked only after China and India. The main fruits produced in the country are bananas, oranges, grapes, pineapples, apples and watermelons, with only pineapple comprising a native Brazilian fruit [3]. In 2019, 50.22% of the world’s fruit production was composed of only five fruits, indicating the low diversity of fruits incorporated in the human diet [4]. The absence of studies and information on native fruits led to the underuse of their potential, as noted for cambuci (*Campomanesia phaea* O. Berg Landrum) and uvaia (*Eugenia pyriformis* Cambess). This indicates the opportunity to increase knowledge concerning these fruits, enabling their dietary insertion to diversify further and enrich the human diet.

Cambuci and uvaia are native Atlantic Rainforest biome fruits belonging to the Myrtaceae family, displaying high production potential, as their attributes follow current trends observed for food consumption in Brazil, such as healthiness and well-being, sustainability and ethics, as well as high sensoriality and pleasure scores. Due to their unique sensory characteristics and high levels of bioactive compounds and antioxidant capacities, cambuci and uvaia have become options to diversify the human diet, as well as an alternative to help the human population consume the minimum daily amount of fruits and vegetables (400 g) suggested by the World Health Organization (WHO) and the Food and Agriculture Organization [1,5,6]. Fruit cultivation can also guarantee biodiversity preservation, create environmental sustainability and improve the lives of small producers through commercial activities employing both fresh fruits or processed products [7].

Cambuci is an ovoid-rhomboidal green fruit (even when ripe) with an astringent acid taste and high pulp yield (approximately 80%). It is consumed almost exclusively in processed form, such as frozen pulp, juices, sweets, jellies and cachaças [5,8]. Uvaia, on the other hand, has a sweet, acidic flavor, is oval, round or piriform, ranges from yellowish to orange, and is consumed both in natura and processed form (such as juices, craft beer, compote, ice cream), in addition, to be used as raw material for cosmetic products, such as shampoo, soaps and moisturizing cream [6,9]. Furthermore, in recent decades, there has been a growing interest in these fruits, either in the development of new products (craft beers, yogurts and nectars) or in the use of fruits as substitute ingredients (replacement of synthetic ingredients with ingredients from native fruits) [10,11].

Previous research has revealed the presence of bioactive compounds (like ascorbic acid, phenolic compounds, volatile compounds tannins and carotenoids), antioxidant capacity (to scavenge synthetic or biological free radicals) and anti-inflammatory activity in both fruits [10,11], although no detailed information on the variability between accessions and localities as performed herein is available.

In this context, the present study aimed to quantify bioactive compounds in uvaia and cambuci accessions obtained from different locations and quantify their antioxidant capacity by employing three analysis methods. It is assumed that both bioactive compounds and antioxidant capacity are higher compared to fruits commonly consumed worldwide, demonstrating the significant potential for cambuci and uvaia production and consumption.

## 2. Materials and Methods

### 2.1. Reagents and Solutions

The following reagents were employed: acetone was acquired from Labsynth (Diadema, SP, Brazil), hexane, ethanol, methanol and methyl tert-butyl ether (MTBE) were acquired from Merck (Darmstadt, Germany), vanillin and sulfuric acid (H_2_SO_4_) were obtained from Dinâmica Química Contemporânea (Diadema, SP, Brazil), and the Folin–Ciocalteu reagent, sodium carbonate Na₂CO₃, dithiothreitol (DTT), metaphosphoric acid, potassium chloride (KCl), Tris (hydroxymethyl) aminomethane, potassium peroxydisulfate, 2,20-azino-bis (3-ethylbenzothiazoline-6-sulfonic acid) diammonium salt (ABTS), monobasic and dibasic potassium phosphate, fluorescein sodium salt, 2,20-azobis(2-methylpropionamidine) dihydrochloride (AAPH), sodium hypochlorite solution (NaOCl) and rhodamine 123 were purchased from Sigma-Aldrich (St. Louis, MO, USA). Gallic acid, ascorbic acid, lutein, zeaxanthin, β-cryptoxanthin, α-carotene β-carotene, catechin, (±)-6-hydroxy-2,5,7,8-tetramethylchromane-2-car- boxylic acid (Trolox) were used as standards and purchased from Sigma-Aldrich (St. Louis, MO, USA). Ultrapure water was obtained from a Millipore Milli-Q System (Merck Millipore, Steinfurt, Germany).

### 2.2. Samples

Cambuci (*Campomanesia phaea* O. Berg Landrum) and uvaia (*Eugenia pyriformis* Cambess) accessions were obtained between October and November 2018 and March and June 2019, respectively. Both fruits were collected when ripe, according to the harvest point established by the Laboratory of Postharvest of Horticultural Products (ESALQ—USP), Piracicaba (SP, Brazil) [12]. Cambucis were collected in the municipalities of Juquitiba, Mogi das Cruzes, Paraibuna, district of Paranapiacaba—Santo André, Riberão Pires, Rio Grande da Serra and Salesópolis, all located in the state of São Paulo—Brazil, on private properties (Table S3); while the uvaias were collected in private fruit farm, in the municipalities of Cabo Verde and Inconfidentes, located in the state of Minas Gerais—Brazil (Table S4). Fully ripe fruits were transported to the Laboratory of Postharvest of Horticultural Products, where homogenized samples containing peel and pulp were prepared and stored in an ultra-freezer at −80 °C until analysis. The samples were incorporated into the laboratory’s native fruit characterization collection.

A total of 51 and 71 cambuci and uvaia accessions were analyzed. Following a Principal Component Analysis (PCA) considered bioactive compounds (vitamin C, phenolic compounds, proanthocyanidins in cambuci and total carotenoids in uvaia) and antioxidant capacity as determined by the ABTS method, 12 cambuci (Table S1) and 19 uvaia (Table S2) accessions were selected for a reactive oxygen species (ROS) deactivation analysis. The uvaia accessions most noteworthy in terms of total carotenoid content (14 accessions) in the PCA (Table S2) were also selected for carotenoid determinations by high-performance liquid chromatography (HPLC).

Of the 12 selected cambuci accessions, one was obtained from the municipality of Rio Grande da Serra, four from Juquitiba and seven from Paraibuna, all in the state of São Paulo—Brazil (Table S3). Of the 19 uvaia accessions selected for the ROS deactivation analysis, 17 were sampled from Cabo Verde and two from Inconfidentes, while 13 accessions were obtained from Cabo Verde and one from Inconfidentes, both in the state of Minas Gerais—Brazil (Table S4) for the HPLC carotenoid analysis (SISGEN record of analyzed accessions: AAEB49D).

### 2.3. Bioactive Compounds

#### 2.3.1. Total Phenolic Compounds

Total phenolic compounds were determined by the Folin–Cicateau spectrophotometric method as described by Woisky and Salatino [13]. Samples (1 g) were extracted with methanol (80% *v*/*v*) and centrifuged at 2795× *g* for 5 min at 4 °C. Then, 0.5 mL of the extract supernatants, 2.5 mL of the Folin–Ciocateau reagent (1:10), and 2.0 mL of a 4% (*w*/*v*) sodium carbonate solution were mixed, followed by homogenization in the dark for 2 h. Absorbances were determined at 740 nm employing a Biochrom Libra S22 spectrophotometer and the results are expressed as mg 100 g^−1^ gallic acid equivalent (GAE) fresh weight (f.w.) using gallic acid as standard.

#### 2.3.2. Ascorbic Acid

Ascorbic acid content was determined according to Pasternak et al. [14], with modifications. The samples (0.1 g) were extracted with metaphosphoric acid (3%) by centrifugation at 7155× *g* for 20 min at 4 °C, filtered through 0.45 μm PTFE filters and mixed with dithiothreitol (DTT), for dehydroascorbic acid reduction. Determinations were performed by reversed-phase HPLC-PDA (ACQ Arc Sys Core 1, Waters), employing an Atlantis dC18 5 μm column (150 mm × 4.6 mm i.d., Waters, Ireland). The mobile phase consisted of a KCl buffer solution (2 mM, pH 2.5) at a 0.8 mL min^−1^ flow rate and detection at 254 nm. Ascorbic acid was identified from the retention times and absorption spectrum similarities with a commercial standard. Results are expressed as mg 100 g^−1^ of vitamin C, f.w.

#### 2.3.3. Total Carotenoid

For the total carotenoid determinations, 1 g of the fresh mass of each fruit was mixed with 9 mL of 100% acetone and centrifuged at 7155× *g* for 20 min at 4 °C. Readings were performed employing a Biochrom Libra S22 spectrophotometer at 661 nm, 644 nm and 470 nm [15]. Total carotenoid contents in the supernatants were calculated according to the following equation:

$$\mathrm{Total}\;\mathrm{carotenoid}=\frac{\left[1000\times A470-\left(1.90\times Ca-63.14\times Cb\right)\right]}{214},$$

where A470 is the absorbance at 470 nm, *Ca* is chlorophyll *a* and *Cb* is chlorophyll *b*, calculated according to the equations below:

Ca=11.24×A661−2.04×A644, Cb=20.13×A644−4194×A661,

where A661 is the absorbance at 661 nm and A644 is the absorbance at 644 nm. The results are expressed as μg g^−1^ of total carotenoids f.w.

#### 2.3.4. Carotenoid Profile and Vitamin a Content

Carotenoid identification and quantification were performed according to Rubio-Diaz et al. [16], with modifications. Samples (1 g) were centrifuged at 7155× *g* for 10 min at 5 °C. The supernatants were then discarded, and the pellets collected. Then, 8 mL of hexane/ethanol/acetone 2:1:1 (*v*/*v*/*v*) were added to the pellet, followed by mixing for 10 min on a TE-140 orbital shaker (Tecnal, Piracicaba, SP, Brazil). Then, 2 mL of ultrapure water was added to the sample, which was mixed for another 10 min. Subsequently, a 3 mL aliquot of the formed lipid fraction was dried under a nitrogen flow. After complete drying, the lipid fraction aliquot was redissolved in 1 mL of methanol/MTBE 60:40 and filtered through 0.22 μm PVDF filters. The entire process was carried out in the dark to avoid carotenoid isomerization and photodegradation. Determinations were performed by reversed-phase HPLC-PDA (ACQ Arc Sys Core 1, Waters) using a C30 YMC Carotenoid 5 μm column (250 mm × 4.6 mml.D., YMC, USA). The mobile phase consisted of (A) 80:15:5 methanol/MTBE/ultrapure water (*v*/*v*/*v*) and (B) 90:7:3 MTBE/methanol/ultrapure water (*v*/*v*/*v*), The mobile phase concentration gradient ranged from 100% A—0 to 3 min, to 80% A and 20% B—18 to 35 min, followed by 5% A and 95% B—45 to 47 min until reaching 100% A—50 to 58 min, at a 1.0 mL/min flow rate. Detections were performed between 340 nm and 500 nm, with typical retention times of 15.47 min for lutein, 17.69 min for zeaxanthin, 26.89 min for β-cryptoxanthin, 38.43 min for α-carotene and 42.20 min for β-carotene. Results are expressed as μg 100 g^−1^ f.w.

Vitamin A content was calculated according to the bioconversion factor proposed by the Institute of Medicine [17]. According to the IOM, 12 µg of β-carotene are equivalent to 1 µg of retinol, while 24 µg of α-carotene and β-cryptoxanthin are equivalent to 1 µg of retinol.

#### 2.3.5. Proanthocyanidins

Proanthocyanidin contents were determined according to Sun, Silva and Spranger [18]. Extracts were obtained by centrifuging 1 g of each sample mixed with methanol (80%) at 1006× *g* for 15 min at 4 °C. Then, 1 mL of the supernatants were mixed with 2.5 mL of vanillin (1%) and 2.5 mL of sulfuric acid (2.5 M). After 15 min in a water bath at 30 °C, readings were taken employing a Biochrom Libra S22 spectrophotometer at 500 nm, and the results are expressed as mg 100 g^−1^ catechin equivalent (CAT) f.w., according to the catechin standard.

### 2.4. Antioxidant Capacity

#### 2.4.1. ABTS

The antioxidant capacity determined by the ABTS^•+^ free radical method was performed according to Al-Duais et al. [19], with modifications. Samples (1 g) were extracted with methanol (80% *v*/*v*) and centrifuged at 2795× *g* for 5 min at 4 °C. The ABTS radical was obtained by reacting 140 nM of potassium persulfate with 7 nM of ABTS^•+^ followed by storage for 16 h in the dark at room temperature. The ABTS^•+^ radical was then diluted with ethanol to an absorbance of 0.70 ± 0.01 at 734 nm. After 6 min of reaction between 300 μL of the extracts and 3000 μL of the ABTS^•+^ radical, absorbances were determined at 734 nm using a Biochrom Libra S22 spectrophotometer. Trolox was used as standard, and the results are expressed as μmol equivalent of Trolox g^−1^ f.w. (μmol g^−1^ TE).

#### 2.4.2. ORAC

Antioxidant capacity determined by the peroxyl (ROO°) elimination followed the Oxygen peroxyl Radical Absorbance Capacity (ORAC) established by Melo et al. [20]. The samples (3 g) were extracted with methanol (80%) and centrifuged at 2795× *g* for 5 min at 4 °C. Then, the supernatant was lyophilized to completely remove the extraction solvent and water from the samples. Then, 20 mg of the freeze-dried samples were suspended in 1 mL of 75 mM phosphate buffer, pH 7.4. Aliquots (30 μL) of the extract were added to a 96-well microplate, followed by the addition of 60 μL of 508.25 nM fluorescein and 110 μL of 76 mM AAPH. A 75 mM phosphate buffer at pH 7.4 was used as blank. The reaction took place at 37 °C, and fluorescence readings were performed every minute for 120 min at an excitation wavelength of 485 nm and emission wavelength 528 nm employing a Molecular Devices LCC microplate reader. Trolox was used as standard, and results are expressed as μmol Trolox equivalents g^−1^ f.w. (μmol g^−1^ TE).

#### 2.4.3. HOCl

The antioxidant capacity determined by the elimination of hypochlorous acid (HOCl) was performed according to Melo et al. [20]. Sample preparation was similar to that performed for the ORAC analysis. Briefly, HOCl was prepared by adjusting the pH of a 1% (m/v) solution of NaOCl to 6.2 with dropwise addition of 10% H_2_SO_4_. he concentration of HOCl was further determined spectrophotometrically at 235 nm using the molar absorption coefficient of 100 M^−1^ cm^−1^ and the proper dilution made in a 100 mM phosphate buffer at pH 7.4. Immediately prior to the analyses, a stock solution of DHR diluted in 1.15 mM of dimethylformamide was used to prepare the working solutions, in phosphate buffer. Extracts (100 μL) at different concentrations, 100 μL of 100 mM phosphate buffer, pH 4.5, 50 μL of DHR and 50 μL of HOCl were then mixed. The analysis was carried out at 37 °C, employing a Molecular Devices LCC microplate reader to determine sample fluorescence at an emission wavelength of 528 nm and excitation wavelength of 485 nm. Results are expressed as EC_50_ (µg mL^−1^) of extract f.w.

### 2.5. Statistical Analyses

All analyses were performed in triplicate and results expressed as means ± standard deviations (SD). Data were submitted to an analysis of variance (ANOVA) and Scott-Knott test (Supplementary Material Tables S1 and S2), through Sisvar software, to classify the investigated fruit accessions, followed by a multivariate Principal Component Analysis (PCA) (Figures S1 and S2), through Statistica 13.5 software, to group them according to the performed analyses. The accessions most noteworthy in terms of phenolic compounds, and ABTS^•+^ antioxidant capacities were then selected for antioxidant capacity determinations by the two other methods and evaluated through an analysis of variance (ANOVA) and Tukey’s test at 5%.

## 3. Results

All accessions for cambuci and uvaia were analyzed for vitamin C content, total phenolic compounds and antioxidant capacity by the ABTS^•+^ method. Furthermore, the cambuci accessions were analyzed in relation to the content of proanthocyanidin and the uvaia accessions in relation to the content of total carotenoids. Based on the results obtained, a PCA was carried out in order to condense the results and correlate them between accessions and analyzes (Figures S1 and S2).

The PCA obtained for cambuci accessions reduced the four variables analyzed into two components, explaining 68.83% of the data variability. Total phenolic compounds and antioxidant capacity by the ABTS^•+^ method were positively correlated, indicating that accessions with high concentration of phenolic compounds tend to present a high antioxidant capacity by the ABTS^•+^ method. Therefore, the accessions that presented correlation with these variables were selected and were grouped in the 2nd quadrant of the graph in the factorial plane for analysis of deactivation of reactive oxygen species (ROS)—12 accessions (Figure S1).

The four variables analyzed for the uvaia accessions were reduced to two main components, after PCA analysis, explaining 73.28% of the data variability. As observed in the PCA of cambuci accessions, there was a positive correlation between total phenolic compounds and antioxidant capacity by the ABTS^•+^ method. Thus, accessions selected were grouped in the 1st quadrant of the graph in the factorial plane for analysis of deactivation of ROS—19 accessions. Furthermore, accessions grouped in the 3rd quadrant of the graph in the factorial plane, were selected for analysis of carotenoids by HPLC—14 accessions (Figure S2).

### 3.1. Bioactive Compounds

The results obtained for the vitamin C content show that both cambuci and uvaia accessions have great potential to be inserted in the population’s diet as native fruits that are a source of vitamin C (Table 1 and Table 2). The highest content detected in cambuci was of 119 mg 100 g^−1^ of vitamin C (Table S1) and in uvaia, 142 mg 100 g^−1^ of vitamin C (Table S2).

The lowest phenolic compound content observed in the cambuci and uvaia accessions was 104 mg 100 g^−1^ of GAE and 89.7 mg 100 g^−1^ of GAE, respectively, and the highest, 173 mg 100 g^−1^ of GAE and 154 mg 100 g^−1^ of GAE, respectively (Table 1 and Table 2). The average phenolic compound concentrations determined for both cambuci (139 mg 100 g^−1^ of GAE) and uvaia (119 mg 100 g^−1^ of GAE) are considered intermediate according to Vasco et al. [21].

Proanthocyanidins are one of the main classes of phenolic compounds in cambuci, responsible for its high astringency and acidic flavor. The maximum, minimum and average content observed was 52.2 mg 100 mg^−1^, 11.5 mg 100 mg^−1^ and 20.6 mg 100 mg^−1^ of catechin, respectively (Table 1).

The average carotenoid contents reported herein was 1.02 × 10^4^ µg 100 g^−1^. It was observed that β-carotene was present at the highest concentrations among the investigated uvaia accessions, proving to be the main pigment present in this fruit, followed by β-cryptoxanthin and zeaxanthin (Table 3). Together, these compounds represent about 99% of the total carotenoids quantified in uvaia. The average zeaxanthin level was 135 µg 100 g^−1,^ while the average lutein level was 39.3 µg 100 g^−1^, ranging from 11.6 µg 100 g^−1^ to 74.1 µg 100 g^−1^. The lutein fraction is the smallest amount of carotenoids quantified in uvaia.

As well as high total carotenoid contents, uvaia also contained high levels of vitamin A (Table 3). α-carotene, β-carotene and β-cryptoxanthin pigments are vitamin precursors, being absorbed and converted into vitamin A in the human body [22]. The results reported herein demonstrate that uvaia can provide an average of 826 µg 100 g^−1^ of fruit RAE.

### 3.2. Antioxidant Capacity

Cambuci displayed a higher mean antioxidant capacity in both the ABTS^•+^ and ORAC analyses, while uvaia presented a higher mean antioxidant capacity in the HOCl scavenging assay (Table 1 and Table 2).

Cambuci ABTS^•+^ antioxidant capacity ranged from 9.23 to 12.2 µmol g^−1^ of TE (Table 1), while uvaia antioxidant capacity by the same method was determined to range from 4.49 to 13.2 µmol g^−1^ of TE (Table 2). Concerning the ORAC method, cambuci exhibited minimum, maximum, and average antioxidant activities of 4.57, 12.5 and 9.17 µmol g^−1^ of TE, respectively. The uvaia exhibited a minimum antioxidant activity of 2.27 µmol g^−1^ of TE, a maximum of 11.7 µmol g^−1^ of TE and an average of 5.92 µmol g^−1^ of TE.

The highest antioxidant capacity value for uvaia determined by the sequestration of hypochlorous acid was 4.87 µg mL^−1^ and the lowest, 11.3 µg mL^−1^. These are unprecedented results, since there are no reports in the literature in this regard. On the other hand, cambuci, showed an average antioxidant capacity by HOCl sequestration of 53.9 µg mL^−1^.

## 4. Discussion

The recommended daily intake of vitamin C for adults is 90 and 75 mg per day for men and women, respectively, which can be obtained mainly from citrus fruits. According to Food Data Central, oranges (*Citrus sinensis*) contain about 60 mg 100 g^−1^ of vitamin C [23,24]. Both cambuci and uvaia (Table 1, Table 2 and Tables S1 and S2) had accessions with levels of vitamin C higher than the National Institutes of Health (NIH) recommendation, as well as orange, strawberry and cantaloupe melons—fruits recognized by the FAO and WHO [25] as rich sources of vitamin C.

Regarding the content of phenolic compounds in cambuci, Spricigo et al. [26] reported an average content of 412.10 mg 100 g^−1^ polyphenols through NMR spectroscopy, higher than in the present study. This difference can be explained by differences in genetics, geographic location and environmental factors, including climate and light incidence, in addition to harvesting during different seasons [27]. For uvaia, levels were close to previous evaluations in the literature. Stafussa et al. [28], for example, reported 132.48 mg 100 g^−1^ of GAE, Rufino et al. [29], 127.0 mg 100 g^−1^ of GAE and da Silva et al. [30], 115.96 mg 100 g^−1^ of GAE.

Healthy and balanced diets have been associated not only with well-being and good body nutrition, but also with the reduction of non-communicable chronic diseases (NCCDs) due to the ingestion of bioactive compounds, such as phytosterols, carotenoids and phenolic compounds [2]. Studies carried out with mice suggest that the phenolic compounds present in both fruits have beneficial actions that exhibit beneficial actions against metabolic complications associated with obesity, oxidative stress modulation, increased antioxidant defense mechanism and attenuate oxidative stress in individuals with NCCD [31,32].

Although considered fruits with intermediate concentrations of phenolic compounds, as mentioned previously, their phenolic compound contents are higher than those of conventional fruits, such as watermelon (55.66 mg 100 g^−1^ of GAE), mango (60.0 mg 100 g^−1^ of GAE), passion fruit (61.0 mg 100 g ^−1^ of GAE), pineapple (69.76 mg 100 g^−1^ of GAE) and melon (69.98 mg 100 g^−1^ of GAE), indicating that both cambuci and uvaia are phenolic compound sources [21,28].

Notwithstanding being responsible for its high astringency and acidic flavor, proanthocyanidin molecules present in cambuci have aroused interest in different study areas, such as nutrition and medicine, due to their high antioxidant activity and potential protective human health effects [33].

Because of this, cambuci accessions containing proanthocyanidin contents equivalent to those detected in mango and Gold kiwi, for example (12.80 and 13.90 mg 100 g^−1^, of total proanthocyanidins, respectively [33] may be more suitable for fresh consumption (accessions 37, 43 and 45), due to their palatability. Fruits belonging to accessions containing higher proanthocyanidin levels (accession 28) may, in turn, be directed to fruit processing, in the form of jellies, liqueurs, and cachaça, for example. Processed products are an interesting alternative for the consumption of cambuci, as even in smaller amounts, they still contain the bioactive compounds and antioxidant activity but without strong astringency or acidity [34].

The average carotenoid contents reported herein in uvaia are about 7-fold higher and 8-fold lower than the total carotenoid contents reported by da Silva et al. [30], of 1306.6 µg 100 g^−1^ and Pereira et al. [35], of 90,933.0 µg 100 g^−1^, respectively. Qualitative and quantitative differences are noted regarding carotenoid content. Various factors, such as climate, geographic location, season, maturation degree, sampled fruit part, postharvest conditions and processing, among others, may affect carotenoid biosynthesis and explain the differences observed for the accessions investigated in the present study with other literature reports [36]. However, the same authors found that uvaias contained β-carotene and β-cryptoxanthin as the most abundant fractions, in agreement with this study [30,35]. Furthermore, the carotenoid contents in uvaias were higher herein than that observed for carrots (8307.0 µg 100 g^−1^), recognized worldwide as a rich source of these compounds [37].

Vitamin A is an essential nutrient for the normal functioning of the visual system, as well as growth and development, and its deficiency causes significant public health problems, especially in developing countries. Stimulating the consumption of foods naturally rich in provitamin A carotenoids, therefore, becomes a way to alleviate vitamin A deficiencies [25]. Knowing that uvaia can provide an average of 826 µg 100 g^−1^ of fruit RAE, the consumption of only 25 and 35 g of uvaia by children younger than six years old and women aged 19 to 65, respectively, is able to provide 100% of the dietary recommendation for this vitamin, aiding in the minimum necessary intake of vitamin A by children and women of reproductive age, the main group affected by Vitamin A deficiency [25].

Unlike provitamin A carotenoids, zeaxanthin is not considered essential. Its importance for eye health has, however, been recognized for a long time. Present in the macula of the eye, zeaxanthin, alongside lutein, can provide protection against potential damage caused by light reaching the retina [38]. The average zeaxanthin levels in uvaia are close to or exceed the levels present in commonly consumed food items, such as cabbage (140 µg of zeaxanthin 100 g^−1^), mango (10 µg of zeaxanthin 100 g^−1^) and papaya (22.1 µg of zeaxanthin 100 g^−1^) [39].

Besides, according to the classification proposed by Britton and Khachik [40], uvaias contain very high β-carotene content (>2000 μg 100 g^−1^), moderate β-cryptoxanthin and zeaxanthin levels (100−500 μg 100 g^−1^) and low α-carotene and lutein content (0−100 μg 100 g^−1^).

Antioxidant capacity was determined from the scavenging of the synthetic radical ABTS^•+^, as well as by the ROS peroxyl (ROO•)–ORAC assay and the hypochlorous acid (HOCl) scavenging assay, the latter two generated during biological processes that take place in the human body. 

The cambuci ABTS^•+^ antioxidant capacity was higher than other fruits such as oranges, guava and passion fruit, which exhibit an antioxidant capacity of approximately 11.93, 5.14 and 3.34 µmol g^−1^ of TE, respectively [28,41]. Castelucci et al. [42] and Stafussa et al. [28] reported cambuci values of 32.06 and 27.80 µmol g^−1^ of TE, higher activities compared to the present findings. Uvaia antioxidant capacity by the same method was similar to other literature reports. As noted for cambuci, uvaias also display a higher ABTS^•+^ antioxidant capacity than commonly consumed fruits, such as tangerine (6.02 µmol g^−1^ of TE), mango (2.7 µmol g^−1^ of TE), pineapple (2.62 µmol g^−1^ of TE) and melon (0.46 µmol g^−1^ of TE) [28,41], suggesting that its human dietary inclusion will help fight free radicals.

About the ORAC method, Tokairin [43] reported 50.59 µmol g^−1^ of TE for cambucis collected in the 2014 harvest and 30.62 µmol g^−1^ of TE for cambucis obtained in the 2015 harvest determined by the same method in the same city, all higher than in the present study. These differences demonstrate how variable antioxidant activity depends on the environment. As with uvaia, they have observed similar results to a previous report by da Silva et al. [44] which, according to the authors, indicate intermediate activity for ROO• ROS deactivation. 

The peroxyl radical present in biological systems is an initial ROS generated at very low concentrations but which undergoes rapid rearrangement and stimulates the chain reaction, mainly in the lipid peroxidation process [45]. The consumption of certain food items has been shown to aid the body’s antioxidant system regarding the elimination of ROO•, and should be encouraged, as lipid peroxidation is associated with cell membrane peroxidation and obesity. Both uvaias and cambucis exhibited an average ORAC antioxidant capacity higher than watermelon (1.42 µmol g^−1^ of TE), papaya (3.00 µmol g^−1^ of TE), cantaloupe melon (93.19 µmol g^−1^ of TE) and pineapple (5.62 µmol g^−1^ of TE), corroborating the antioxidant capacity of these native fruits [46].

Like the peroxyl radical, the reactive HOCl species can also be found in human biological systems. The antioxidant capacity values determined by the sequestration of hypochlorous acid are expressed in EC50 (amount of sample necessary to deactivate 50% of the reactive HOCl species), in which the lower the result, the greater the antioxidant activity of the investigated fruits. 

The high antioxidant capacity value determined by this method to uvaia demonstrates the significant potential of this fruit in eliminating this ROS, which contributes to the development of some NCCDs, such as atherosclerosis, degenerative diseases and certain types of cancer [47]. These are unprecedented results considering that no literature reports in this regard are available. Other species belonging to the Eugenia genus, such as grumixama, cereja do Rio-Grande, araçá-piranga and pêssego-do-mato, exhibit antioxidant capacities of about 5-, 7-, 13- and 17-fold lower, respectively, than the average observed for uvaia [48]. To cambuci, HOCl sequestration results are available in the literature, with a reported antioxidant capacity of 74.14 µg mL^−1^ [47], close to the average observed herein. When compared to the general average observed for uvaia, cambuci displays about sixfold less antioxidant capacity and higher when compared with the Trolox standard (EC50 134 µg mL^−1^) [49].

## 5. Conclusions

The findings reported here reveal that cambuci and uvaias as fruits presenting higher bioactive compounds and higher antioxidant capacities than other widely consumed fruits around the world. The unprecedented results on the antioxidant capacity by the sequestration of hypochlorous acid for uvaia validate this idea. The variability found in the results for vitamin C in both fruits and for proanthocyanidins in cambuci accessions indicates a potential to encourage the consumption of these fruits widely, both in natura and as a raw material for industrialized products. Studies on the in vivo antioxidant capacity, bioavailability and bioaccessibility of bioactive compounds and even epidemiological studies with the inclusion of dietary cambuci and uvaia thus become feasible in view of the positive results reported herein. Finally, it is assumed that the regular consumption of these fruits may lead to beneficial health effects, in addition to diversifying the human diets and assisting in the minimum daily recommended fruit consumption.

## Figures and Tables

**Table 1:** Vitamin C, proanthocyanidin and phenolic compound contents and antioxidant activity of the 12 cambuci accessions investigated in the present study.

Accession	Variables											
	VIT.C		PA		CF		ABTS		ORAC		HOCl	
21	62.0 ± 4.73	b	26.3 ± 1.75	b	111 ± 11.88	fg	12.2 ± 0.53	a	8.77 ± 0.69	ab	47.5 ± 5.14	bc
23	26.0 ± 2.56	f	17.8 ± 3.45	cde	157 ± 7.17	abc	9.42 ± 0.59	a	4.57 ± 0.66	b	61.2 ± 3.72	abc
25	47.6 ± 2.61	cd	15.4 ± 3.81	cde	104 ± 10.3	g	10.6 ± 2.45	a	8.00 ± 0.49	ab	50.2 ± 2.60	abc
27	80.4 ± 0.96	a	22.8 ± 1.66	bc	134 ± 4.36	de	10.1 ± 1.66	a	10.9 ± 1.45	ab	62.9 ± 7.82	ab
28	74.8 ± 3.08	a	52.2 ± 3.00	a	122 ± 7.23	efg	9.27 ± 0.97	a	12.5 ± 2.48	a	64.7 ± 4.50	a
37	44.3 ± 1.65	d	11.5 ± 2.20	e	138 ± 2.75	bcde	12.0 ± 0.45	a	11.3 ± 2.43	ab	67.3 ± 4.50	a
40	52.3 ± 3.34	c	21.4 ± 0.84	bcd	127 ± 3.01	ef	9.74 ± 2.43	a	11.0 ± 1.80	ab	62.5 ± 13.53	ab
41	49.7 ± 0.97	cd	15.4 ± 1.14	cde	155 ± 13.9	abcd	11.4 ± 1.30	a	8.85 ± 2.78	ab	39.9 ± 2.57	a
42	32.4 ± 4.65	ef	14.2 ± 2.59	de	157 ± 6.11	abc	9.23 ± 2.72	a	11.3 ± 6.49	ab	46.7 ± 3.06	abc
43	35.9 ± 3.03	e	12.9 ± 1.08	e	173 ± 5.20	a	11.2 ± 1.26	a	6.93 ± 1.27	ab	52.2 ± 9.17	abc
45	36.6 ± 3.26	e	12.7 ± 2.70	e	159 ± 3.77	ba	11.8 ± 0.67	a	9.28 ± 2.28	ab	47.2 ± 5.48	abc
49	24.8 ± 2.11	f	26.6 ± 3.66	b	136 ± 3.56	cde	9.67 ± 0.86	a	6.58 ± 1.75	ab	50.7 ± 0.82	abc
Overall means	47.2		20.6		139		10.5		9.17		53.9	
CV (%)	5.46		12.65		5.42		12.5		28.4		13.4	

Each value is expressed as means (triplicate) ± standard deviation (SD). Means followed by the same letter do not differ statistically from each other. Vitamin C (VIT.C): mg 100 g^−1^ VIT.C; proanthocyanidin (PA): mg 100 g^−1^ CAT; phenolic compounds (CF): mg 100 g^−1^ GAE; antioxidant activity by ABTS^•+^ method: µmol g^−1^ of Trolox equivalent; ORAC antioxidant activity: µmol g^−1^ Trolox equivalent; antioxidant activity by HOCl sequestration: EC_50_ µg mL^−1^; CV (%): coefficient of variation. All data are significant at the 5% probability level (*p* < 0.05) (one-way ANOVA and Tukey’s test at the 5% level).

**Table 2:** Vitamin C and phenolic compound contents and antioxidant activity of the 19 uvaia accessions investigated in the present study.

Accession	Variables									
	VIT.C		CF		ABTS		ORAC		HOCl	
5	88.0 ± 4.09	de	136 ± 4.36	bc	8.04 ± 0.92	def	8.42 ± 1.67	ab	8.76 ± 0.84	abcd
7	89.4 ± 3.91	de	97.5 ± 2.75	hi	6.80 ± 0.69	efghi	7.16 ± 0.63	ab	9.81 ± 1.24	ab
17	102 ± 1.07	c	107 ± 7.44	fgh	5.29 ± 2.79	ijk	8.58 ± 1.69	ab	11.3 ± 1.67	a
18	128 ± 1.48	a	100 ± 2.36	hi	7.94 ± 4.22	defg	8.67 ± 1.49	ab	5.06 ± 3.17	cd
22	62.9 ± 2.05	i	124 ± 3.19	cde	11.5 ± 1.64	b	6.09 ± 1.48	ab	9.44 ± 1.54	ab
25	112 ± 2.57	b	140 ± 3.12	b	6.04 ± 0.60	hijk	3.43 ± 1.45	b	7.07 ± 1.16	bcd
33	135 ± 2.78	a	122 ± 1.70	cde	8.10 ± 4.63	de	5.49 ± 1.38	ab	6.17 ± 1.42	bcd
34	75.6 ± 2.88	fgh	121 ± 5.92	de	8.32 ± 4.74	de	3.36 ± 0.95	b	7.28 ± 1.23	bcd
38	103 ± 4.01	c	102 ± 4.33	ghi	7.33 ± 2.16	efgh	4.59 ± 1.06	b	7.32 ± 1.02	bcd
43	77.9 ± 3.68	fgh	154 ± 6.95	a	9.07 ± 2.67	cd	11.7 ± 6.97	a	7.72 ± 0.47	abcd
45	77.3 ± 2.16	fgh	118 ± 3.21	ef	10.4 ± 5.11	bc	7.90 ± 3.25	ab	9.84 ± 0.89	ab
50	82.2 ± 1.78	ef	116 ± 5.03	efg	4.49 ± 2.11	k	3.09 ± 0.42	b	5.05 ± 0.32	d
52	92.8 ± 1.72	d	89.7 ± 5.29	i	10.9 ± 4.31	b	8.41 ± 2.21	ab	9.29 ± 0.98	ab
54	105 ± 3.97	bc	122 ± 2.09	de	13.2 ± 3.63	a	4.61 ± 1.33	b	8.28 ± 1.62	abcd
57	105 ± 0.34	fgh	102 ± 2.53	ghi	6.43 ± 2.59	fghij	5.68 ± 1.89	ab	6.79 ± 0.63	bcd
58	79.8 ± 0.67	fg	120 ± 6.37	ef	5.38 ± 2.18	ijk	2.27 ± 1.03	b	9.03 ± 0.91	abc
59	72.6 ± 2.90	gh	139 ± 0.62	b	6.40 ± 0.56	ghij	5.72 ± 0.84	ab	9.65 ± 0.78	ab
66	70.7 ± 1.32	hi	122 ± 9.20	cde	5.15 ± 1.26	jk	4.25 ± 0.24	b	4.87 ± 0.28	d
68	50.9 ± 3.09	j	134 ± 9.19	bcd	6.16 ± 0.88	hij	3.07 ± 0.20	b	5.89 ± 0.53	bcd
Overall means	88.6		119		7.73		5.92		7.82	
CV (%)	2.96		3.79		40.2		36.9		16.5	

Each value is expressed as means (triplicate) ± standard deviation (SD). Means followed by the same letter do not differ statistically from each other. Vitamin C (VIT.C): mg 100 g^−1^ VIT.C; phenolic compounds (CF): mg 100 g^−1^ GAE; antioxidant activity by ABTS^•+^ method: µmol g^−1^ of Trolox equivalent; ORAC antioxidant activity: µmol g^−1^ Trolox equivalent; antioxidant activity by HOCl sequestration: EC_50_ µg mL^−1^; CV (%): coefficient of variation. All data are significant at the 5% probability level (*p* < 0.05) (one-way ANOVA and Tukey’s test at the 5% level).

**Table 3:** Carotenoid content of the 14 uvaia accessions investigated in the present study.

Accession	Carotenoids (µg 100 g^−1^)						RAE ^1^ (µg 100 g^−1^)
	Lutein	Zeaxanthin	β-Cryptoxanthin	α-Carotene	β-Carotene	Total Carotenoids	Vitamin A
Peak No.	1	2	3	4	5		
RT (Min)	15.9	18.1	28.2	40.5	42.9		
3	74.1 ± 3.29	12.0 ± 0.55	408 ± 6.84	102 ± 2.80	7.63 × 10^3^ ± 56.96	8.22 × 10^3^ ± 70.46	657 ± 5.15
4	68.7 ± 2.95	48.9 ± 1.41	249. ± 7.16	94.0 ± 0.23	5,79 × 10^3^ ± 51.96	6.25 × 10^3^ ± 63.70	497 ± 4.64
6	40.1 ± 1.40	111 ± 1.63	648 ± 5.15	62.8 ± 3.49	1.05 × 10^4^ ± 55.41	1.14 × 10^4^ ± 67.07	911 ± 4.98
9	38.2 ± 2.10	46.56 ± 1.55	452 ± 7.83	131 ± 5.01	2.86 × 10^4^ ± 39.41	2.92 × 10^4^ ± 55.90	2.40 × 10^3^ ± 3.82
24	35.7 ± 2.25	294 ± 5.04	480 ± 7.69	26.7 ± 2.81	8.06 × 10^3^ ± 45.98	8.90 × 10^3^ ± 63.76	693 ± 4.27
26	40.4 ± 5.12	279 ± 3.63	392 ± 2.34	68.9 ± 2.91	1.38 × 10^4^ ± 33.26	1,46 × 10^4^ ± 47.26	1.17 × 10^3^ ± 2.99
28	22.2 ± 0.34	77.1 ± 1.04	359 ± 3.85	63.9 ± 4.27	8.82 × 10^3^ ± 21.82	9.34 × 10^3^ ± 31.33	753 ± 2.16
36	39.7 ± 3.58	127 ± 5.36	274 ± 3.87	43.3 ± 0.56	7.02 × 10^3^ ± 41.07	7.50 × 10^3^ ± 54.44	598 ± 3.61
37	11.6 ± 0.62	208 ± 3.32	92.3 ± 5.37	12.9 ± 1.91	3.96 × 10^3^ ± 29.01	4.29 × 10^3^ ± 40.23	335 ± 2.72
39	57.6 ± 4.58	135 ± 3.64	222 ± 3.66	74.3 ± 6.12	5.61 × 10^3^ ± 36.61	6.10.10^3^ ± 54.60	481 ± 3.46
40	15.3 ± 2.48	153 ± 4.67	262 ± 5.60	39.9 ± 6.62	5.58 × 10^3^ ± 52.84	6.05 × 10^3^ ± 72.20	478 ± 4.91
41	72.6 ± 1.77	56.9 ± 3.84	439 ± 2.18	113 ± 0.91	1.77 × 10^4^ ± 75.49	1.84 × 10^4^ ± 84.20	1.50 × 10^3^ ± 6.42
46	23.2 ± 1.65	237 ± 3.87	273 ± 0.88	22.9 ± 1.53	5.36 × 10^3^ ± 7.57	5.91 × 10^3^ ± 15.50	459 ± 0.73
55	13.7 ± 2.52	111 ± 0.52	189 ± 1.75	42.3 ± 3.40	7.32 × 10^3^ ± 20.22	7.68 × 10^3^ ± 28.42	620 ± 1.90
Overal means	39.3	135	338	64.1	9.71 × 10^3^	1.02 × 10^4^	826
CV (%)	7.14	2.25	1.20	5.31	0.40	-	-

Each value is expressed as mean (triplicate) ± standard deviation (SD). ¹ RAE: retinol activity equivalent.

## Data Availability

Data available in a publicly accessible repository that does not issue DOIs. Publicly available datasets were analyzed in this study. This data can be found here: https://drive.google.com/drive/u/1/folders/1dhPasIpGC8_Iall4b5Dd8lF1x55_8RU1 (accessed on 25 July 2022).

## Supplementary Materials

The following supporting information can be downloaded at: https://www.mdpi.com/article/10.3390/foods11172612/s1, Figure S1: Principal component analysis (PCA) for the 51 cambuci accessions investigated herein indicating accession factorial plane projections. Figure S2: Principal component analysis (PCA) for the 71 uvaia accessions investigated herein, indicating accession factorial plane projections. Table S1: Vitamin C content, proanthocyanidins, total phenolic compounds and antioxidant capacity determined by the ABTS•+ method for the 51 cambuci accessions investigated herein (means followed by standard deviations). Table S2: Vitamin C content, total carotenoids, total phenolic compounds and antioxidant capacity determined by the ABTS^•+^ method of the 71 uvaia accessions investigated herein (means followed by standard deviations). Table S3: Accessions and sampling municipalities of the 51 cambuci accessions investigated herein. Table S4: Accessions and sampling municipalities of the 71 uvaia accessions investigated herein. Reference [50] is cited in the supplementary materials.
