# Supplementary material for: Bioactive Compounds and In Vitro Antioxidant Capacity of Cambuci and Uvaia: An Extensive Description of Little-Known Fruits from the Myrtaceae Family with High Consumption Potential
Source: Foods. 2022 Aug 29;11(17):2612. doi: 10.3390/foods11172612 (PMC9455873; doi:10.3390/foods11172612)
Supplement: Supplementary file 1 [file foods-11-02612-s001.zip › foods-1862206-supplementary.pdf]

Supplemetary

Bioactive compounds and in vitro antioxidant capacity of cambuci and uvaia: an extensive description of little known fruits from the Myrtaceae family with high consumption potential

Isabela Barroso Taver [1], Poliana Cristina Spricigo [1], Horst Bremer Neto[1], Severino Matias de Alencar[1], Adna Prado Massarioli[1] and Angelo Pedro Jacomino [1]

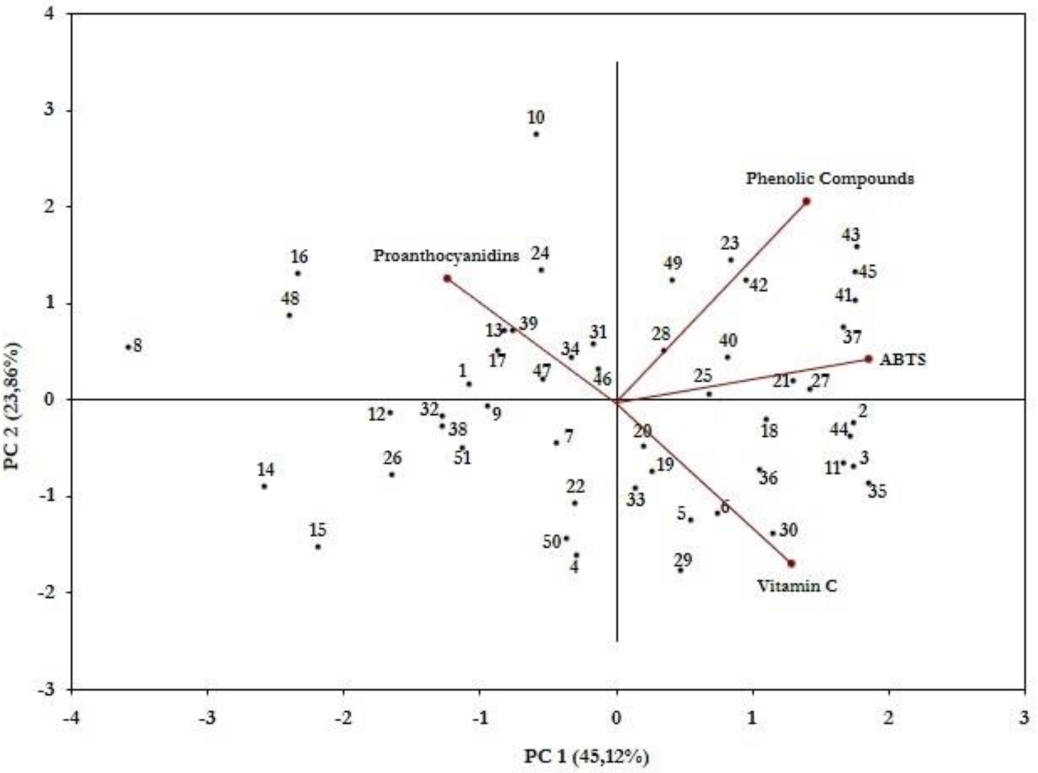

Figure S1. Principal component analysis (PCA) for the 51 cambuci accessions investigated herein indicating accession factorial plane projections.

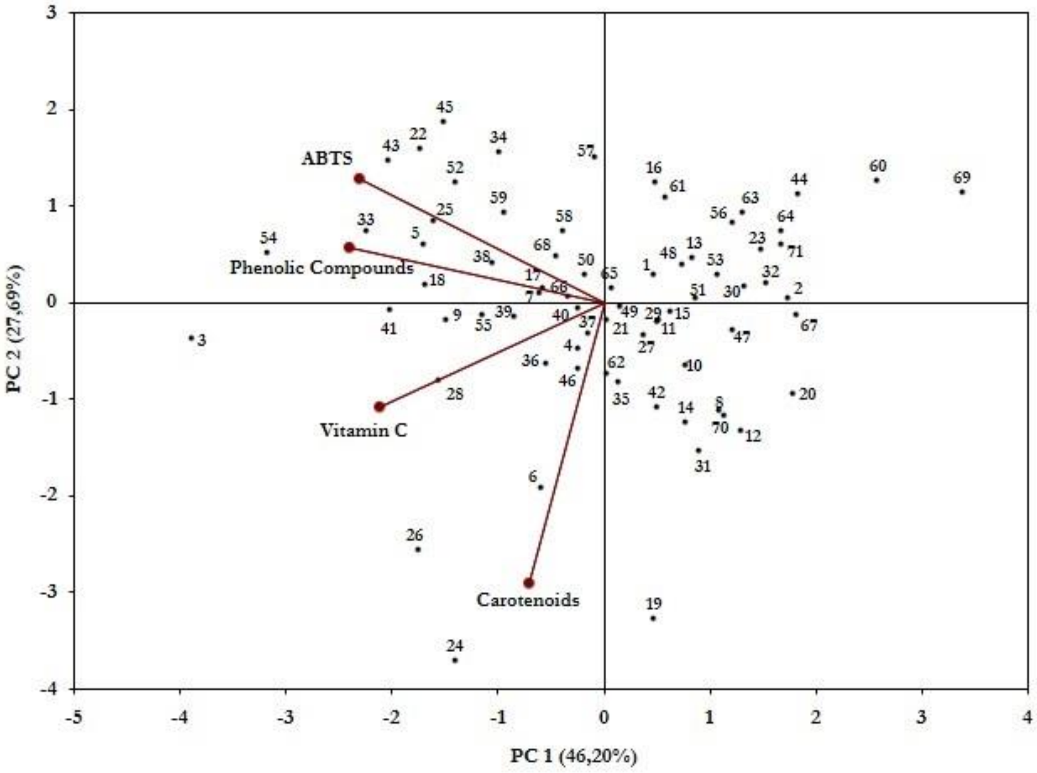

Figure S2. Principal component analysis (PCA) for the 71 uvaia accessions investigated herein indicating accession factorial plane projections.

Table S1. Vitamin C content, proanthocyanidins, total phenolic compounds and antioxidant capacity determined by the ABTS•+ method for the 51 cambuci accessions investigated herein (means followed by standard deviations).

Accessions	Variables							
	VIT.C		PA		CF		ABTS	
1	37.5±4.21	j	26.9±0.41	g	109±11.6	e	4.86±0.41	d
2	98.5±3.12	c	25.44±1.53	g	129±14.0	d	10.8±1.42	a
3	111±1.26	b	14.0±2.20	k	136±3.45	c	8.94±0.39	b
4	73.2±1.60	f	11.3±2.92	k	63.1±13.9	h	6.91±0.79	c
5	78.0±1.62	e	18.9±0.95	i	68.4±12.2	h	10.1±1.47	b
6	96.3±3.94	c	31.0±1.88	f	75.5±4.82	g	10.4±0.26	a
7	11.0±0.75	n	17.4±2.44	j	42.8±5.32	i	11.3±0.60	a
8	14.8±5.26	m	79.4±1.60	b	56.1±10.3	h	3.51±0.79	e
9	24.1±3.09	l	24.2±1.32	h	80.1±4.73	g	7.35±1.15	c
10	23.7±5.10	l	83.1±2.07	b	151±13.5	b	9.57±1.40	b
11	118±3.72	a	33.6±3.63	f	118±6.64	d	10.6±0.96	a
12	23.3±0.98	l	40.9±1.80	e	58.5±4.76	h	7.11±0.77	c
13	21.5±2.04	l	30.0±2.30	f	112±10.39	e	6.77±0.80	c
14	23.8±1.55	l	31.8±0.12	f	40.3±3.42	i	3.88±0.19	e
15	78.7±3.16	e	54.3±1.28	c	36.2±13.7	i	4.27±0.82	d
16	45.4±4.44	i	112±0.20	a	74.4±13.5	g	8.02±0.73	c
17	17.1±2.30	m	33.1±1.46	f	90.4±7.58	f	8.12±0.77	c
18	64.2±4.64	g	18.6±1.44	i	105±13.5	e	11.0±1.73	a
19	73.3±1.97	f	20.0±2.09	i	94.5±10.7	f	8.04±1.06	c
20	51.7±0.83	h	13.6±2.91	k	92.1±9.79	f	8.62±0.87	b
21	62.0±4.73	g	26.3±1.75	g	111±11.88	e	12.2±0.53	a
22	51.1±0.68	h	22.8±1.77	h	48.5±4.18	i	9.70±0.59	b
23	26.0±2.56	l	17.8±3.45	j	157±7.17	b	9.42±0.43	b
24	29.0±1.97	k	54.5±3.04	c	118.3±9.16	d	8.92±0.68	b
25	47.6±2.61	i	15.4±3.81	j	104±10.3	e	10.6±1.60	a
26	32.0±1.12	k	23.0±1.39	h	59.6±5.12	h	5.24±0.54	d

27	80.4±0.96	d	22.8±1.66	h	134±4.36	c	10.1±0.68	b
28	74.8±3.08	f	52.2±3.00	d	122±7.23	d	9.27±0.14	b
29	97.3±4.48	c	16.0±1.17	j	67.9±4.79	h	8.53±0.84	b
30	81.8±3.22	d	10.5±0.42	l	69.9±5.18	h	11.4±0.88	a
31	50.3±1.98	j	56.7±4.38	c	91.7±4.54	f	10.7±0.85	a
32	25.8±1.43	l	31.3±3.22	f	68.8±4.84	h	7.13±0.93	c
33	38.6±1.21	j	5.72±1.33	m	61.2±7.18	h	10.1±1.35	b
34	28.7±0.81	k	31.3±2.01	f	96.2±3.26	f	9.08±0.84	b
35	119±5.19	a	29.1±2.97	f	111±10.33	e	11.3±0.75	a
36	84.8±4.74	d	13.0±1.31	k	111±12.60	e	9.03±1.24	b
37	44.3±1.65	i	11.5±2.20	k	138±2.75	c	12.0±0.45	a
38	6.06±2.71	o	16.5±2.13	j	59.4±4.00	h	7.55±0.50	c
39	16.7±2.65	m	35.0±1.62	f	96.9±3.36	f	8.40±0.71	b
40	52.3±3.34	h	21.4±0.84	i	127±3.01	d	9.74±1.16	b
41	49.7±0.97	h	15.4±1.14	j	155±13.95	b	11.4±0.45	a
42	32.4±4.65	k	14.2±2.59	k	157±6.11	b	9.23±0.47	b
43	35.9±3.03	j	12.9±1.08	k	173±5.20	a	11.2±0.14	a
44	86.1±4.62	d	10.1±3.10	l	128±9.42	d	10.3±0.72	a
45	36.6±3.26	j	12.7±2.70	k	159±3.77	b	11.8±0.57	a
46	44.0±1.74	i	26.7±1.08	g	114±13.2	e	7.74±0.61	c
47	33.4±2.35	j	26.2±2.26	g	100±4.11	e	7.46±0.68	c
48	15.5±1.79	m	47.8±2.11	d	108±8.52	e	2.79±1.55	e
49	24.8±2.11	l	26.6±3.66	g	136±3.56	c	9.67±0.12	b
50	67.9±2.89	g	10.1±1.47	l	69.3±4.43	h	6.53±1.95	a
51	38.0 ±4.32	j	16.0±6.34	j	87.4±7.24	f	4.86±1.49	d
Means	50.9		28.4		99.5		8.69	
CV (%)	5.91		8.34		8.61		10.2	

Each value is expressed as means (triplicate) ± standard deviations (SD). Means followed by the same letter do not differ statistically from each other. Vitamin C (VIT.C): mg 100 g^{-1} VIT.C; proanthocyanidin (PA): mg 100 g^{-1} CAT; phenolic compounds (CF): mg 100 g^{-1} GAE; antioxidant activity determined by the $ABTS^{\bullet+}$ method: μmol g^{-1} of trolox; ORAC antioxidant activity: $μmol^{-1}$ trolox; antioxidant activity by HOCl sequestration: EC_{50} mg mL^{-1}; CV (%): coefficient of variation. All data are significant at the 5% probability level ($p < .05$) (one-way ANOVA and Scott-Knott test at the 5% level).

Table S2. Vitamin C content, total carotenoids, total phenolic compounds and antioxidant capacity determined by the ABTS$^{•+}$ method of the 71 uvaia accessions investigated herein (means followed by standard deviations).

Accessions	Variables							
	VIT.C		CA		CF		ABTS	
1	71.7±2.05	k	37.5±2.67	g	109.1±8.63	e	3.45±0.93	j
2	66.4±1.33	l	35.4±1.40	g	70.4±8.71	h	2.31±0.41	l
3	135±3.94	b	56.6±1.18	g	186.2±7.68	a	7.92±0.79	e
4	92.3±3.16	h	50.1±3.15	e	92.4±7.66	f	5.39±0.13	h
5	88.0±4.09	h	44.7±4.39	f	136±4.36	c	8.04±0.92	e
6	86.0±1.07	h	76.1±5.95	b	104.2±5.96	e	4.78±0.31	h
7	89.4±3.91	h	45.2±4.49	f	97.5±2.75	f	6.80±0.69	f
8	72.2±2.80	j	56.8±2.66	d	77.8±4.55	g	2.77±0.58	k
9	111±2.30	e	43.9±2.39	f	107±9.01	e	6.91±0.44	f
10	84.5±3.52	h	49.5±3.00	e	64.3±8.64	i	4.49±0.77	h
11	89.9±3.24	h	41.0±2.29	f	78.8±6.81	g	4.24±0.52	i
12	73.3±1.72	j	59.4±2.95	d	63.4±4.02	i	2.99±0.73	k
13	66.0±3.06	l	38.0±2.33	g	74.2±5.46	h	5.51±0.38	h
14	80.2±3.67	i	59.4±2.50	d	71.3±2.30	h	3.76±0.15	j
15	107±2.96	e	33.0±1.73	h	61.2±7.26	i	4.18±0.93	i
16	91.0±2.16	h	17.6±0.10	k	81.3±4.58	g	5.06±0.76	h
17	102±1.07	f	38.1±0.55	g	107±7.44	e	5.29±0.59	h
18	128±1.48	c	38.7±1.28	g	100±2.36	e	7.94±0.91	e
19	80.0±3.85	i	95.4±4.34	a	66.2±6.20	h	3.73±0.32	j
20	70.4±2.84	k	50.0±2.08	e	61.1±6.11	i	1.91±0.04	l
21	63.0±1.86	l	50.6±5.37	e	128±3.40	d	3.68±0.55	j
22	62.9±2.05	l	41.4±2.24	f	124±3.19	d	11.5±0.55	b
23	53.1±0.68	m	34.0±1.29	h	85.0±4.27	g	3.27±0.53	j
24	142±3.64	a	90.5±4.26	a	104±6.65	e	0.59±0.39	k
25	112±2.57	e	29.4±1.41	i	140±3.12	c	6.04±0.08	g
26	97.7±3.61	g	92.1±3.68	a	126±3.65	d	6.12±0.52	g

27	74.8±5.40	j	51.2±3.97	e	77.1±6.93	g	5.72±0.56	g
28	112±3.25	e	58.0±2.56	d	113.8±3.30	d	6.74±0.73	f
29	54.4±2.82	m	50.8±2.80	e	115±1.43	d	3.65±0.68	j
30	66.0±1.57	l	35.7±1.89	g	86.6±3.30	f	2.59±0.35	k
31	92.3±2.43	h	57.5±2.82	d	73.9±4.18	h	2.10±0.68	l
32	75.2±3.03	j	30.9±0.84	i	73.5±5.97	h	2.31±0.36	l
33	135±2.78	b	29.8±1.28	i	122±1.70	d	8.10±0.57	e
34	75.6±2.88	j	29.5±1.45	i	121±5.92	d	8.32±0.36	e
35	121±3.15	d	43.4±4.57	f	63.9±2.14	i	4.25±0.11	i
36	109±1.45	e	48.8±3.32	e	92.9±3.40	f	5.24±0.35	h
37	94.8±3.35	g	45.9±1.34	f	92.1±1.84	f	5.02±0.35	h
38	103±4.01	f	38.7±1.99	g	102±4.33	e	7.33±0.69	f
39	100±2.20	f	42.3±2.66	f	127±6.04	d	5.25±0.63	h
40	98.0±2.69	g	40.3±1.88	f	101±5.70	e	4.66±0.49	h
41	110±3.90	e	47.6±2.50	e	151±1.65	b	6.08±0.08	g
42	75.4±3.07	j	59.9±3.14	d	86.8±2.39	f	3.92±0.33	i
43	77.9±3.68	i	36.6±2.59	g	154±6.95	b	9.07±0.29	d
44	34.8±0.62	o	31.9±3.96	h	63.5±2.96	i	5.40±0.48	h
45	77.3±2.16	i	29.7±3.13	i	118±3.21	d	10.4±0.41	c
46	90.5±1.48	h	50.2±1.33	e	125±5.11	d	2.75±0.46	k
47	71.0±3.08	k	43.4±3.26	f	75.0±8.80	h	3.21±0.65	j
48	65.7±1.10	l	37.1±2.69	g	95.5±3.17	f	4.08±0.12	i
49	88.9±3.82	h	40.9±2.83	g	89.5±7.33	f	4.76±0.58	h
50	82.2±1.78	i	38.7±3.03	g	116±5.03	d	4.49±0.34	h
51	95.4±0.53	g	33.2±0.76	h	61.4±5.43	i	4.17±0.45	i
52	92.8±1.72	h	35.2±1.00	g	89.7±5.29	f	10.9±0.64	c
53	77.9±3.16	i	33.7±0.31	h	68.7±6.68	h	4.18±0.69	i
54	105±3.97	f	55.7±6.94	d	122±2.09	d	13.2±0.70	a
55	74.6±3.92	j	56.8±3.00	d	129±7.34	d	6.71±0.71	f
56	70.9±2.03	k	25.4±4.58	j	79.1±4.45	g	3.71±0.16	j
57	105±0.34	i	22.0±2.32	j	102±2.53	e	6.43±0.28	f

58	79.8±0.67	i	34.4±3.03	h	120±6.37	d	5.38±0.50	h
59	72.6±2.90	j	37.7±1.31	g	139±0.62	c	6.40±0.12	f
60	32.4±0.42	o	22.1±2.12	j	69.7±6.12	h	2.56±0.54	k
61	51.8±2.51	m	32.3±2.24	h	108±2.79	e	5.01±0.89	h
62	69.4±3.80	k	57.3±1.36	d	121±6.81	d	3.41±0.75	j
63	57.2±2.02	m	26.9±0.36	j	91.7±9.05	f	3.33±0.36	j
64	41.9±1.57	n	33.5±3.35	h	84.8±7.08	g	3.48±0.55	j
65	64.3±3.30	l	47.1±0.45	e	107±2.80	e	5.38±0.21	h
66	70.7±1.32	k	48.2±2.17	e	122±9.20	d	5.15±0.77	h
67	28.9±3.87	o	52.5±0.85	e	73.9±8.30	h	4.05±0.13	i
68	50.9±3.09	m	49.5±3.46	e	134±9.19	c	6.16±0.37	g
69	19.8±1.59	p	26.5±3.24	j	48.9±6.61	i	2.14±0.40	l
70	62.7±1.17	l	64.2±3.46	c	54.0±6.70	i	5.07±0.58	h
71	50.6±0.10	m	34.1±0.69	h	69.1±3.57	h	4.09±0.56	i
Means	81.0		44.3		97.8		5.2	
CV (%)	4.25		6.01		8.33		8.94	

Each value is expressed as means (triplicate) ± standard deviation (SD). Means followed by the same letter do not differ statistically from each other. Vitamin C (VIT.C): mg 100 g^{-1} VIT.C; total carotenoids (CA): µg g^{-1}; phenolic compounds (CF): mg 100 g^{-1} GAE; antioxidant activity by ABTS ^{o}+ method: µmol g^{-1} of trolox; ORAC antioxidant activity: µmol g^{-1} trolox; antioxidant activity by HOCl sequestration: EC_{50} mg mL^{-1}; CV (%): coefficient of variation. All data are significant at the 5% probability level ($p < .05$) (one-way ANOVA and Scott-Knott test at the 5% level).

Table S3. Accessions and sampling municipalities of the 51 cambuci accessions investigated herein.

Accessions	Cambuci		Accessions	Cambuci	
	Municipalities	Climate[1]		Municipalities	Climate[1]
1	Paraibuna	Cfb	27	Paraibuna	Cfb
2	Paraibuna	Cfb	28	Paraibuna	Cfb
3	Paraibuna	Cfb	29	Paraibuna	Cfb
4	Paraibuna	Cfb	30	Paraibuna	Cfb
5	Paraibuna	Cfb	31	Ribeirão Pires	Cfa
6	Paraibuna	Cfb	32	Ribeirão Pires	Cfa
7	Paraibuna	Cfb	33	Paranapiacaba	Cfa
8	Mogi das Cruzes	Cwa	34	Ribeirão Pires	Cfa
9	Mogi das Cruzes	Cwa	35	Paraibuna	Cfb
10	Mogi das Cruzes	Cwa	36	Paraibuna	Cfb
11	Mogi das Cruzes	Cwa	37	Paraibuna	Cfb
12	Mogi das Cruzes	Cwa	38	Mogi das Cruzes	Cwa
13	Mogi das Cruzes	Cwa	39	Paranapiacaba	Cfa
14	Juquitiba	Cfa	40	Juquitiba	Cfa
15	Juquitiba	Cfa	41	Juquitiba	Cfa
16	Juquitiba	Cfa	42	Juquitiba	Cfa
17	Juquitiba	Cfa	43	Juquitiba	Cfa
18	Juquitiba	Cfa	44	Paraibuna	Cfb
19	Juquitiba	Cfa	45	Paraibuna	Cfb
20	Rio Grande da Serra	Cfa	46	Paraibuna	Cfb
21	Rio Grande da Serra	Cfa	47	Paraibuna	Cfb
22	Rio Grande da Serra	Cfa	48	Paraibuna	Cfb
23	Paraibuna	Cfb	49	Paraibuna	Cfb
24	Paraibuna	Cfb	50	Paraibuna	Cfb
25	Paraibuna	Cfb	51	Paraibuna	Cfb
26	Salesópolis	Cfa			

[1] Köppen climate classification [50].

Table S4. Accessions and sampling municipalities of the 71 uvaia accessions investigated herein.

Accessions	Uvaia		Accessions	Uvaia	
	Municipalities	Climate[1]		Municipalities	Climate[1]
1	Cabo Verde	Cwa	37	Cabo Verde	Cwa
2	Cabo Verde	Cwa	38	Cabo Verde	Cwa
3	Cabo Verde	Cwa	39	Cabo Verde	Cwa
4	Cabo Verde	Cwa	40	Cabo Verde	Cwa
5	Cabo Verde	Cwa	41	Cabo Verde	Cwa
6	Cabo Verde	Cwa	42	Cabo Verde	Cwa
7	Cabo Verde	Cwa	43	Cabo Verde	Cwa
8	Cabo Verde	Cwa	44	Cabo Verde	Cwa
9	Cabo Verde	Cwa	45	Cabo Verde	Cwa
10	Cabo Verde	Cwa	46	Cabo Verde	Cwa
11	Cabo Verde	Cwa	47	Cabo Verde	Cwa
12	Cabo Verde	Cwa	48	Cabo Verde	Cwa
13	Cabo Verde	Cwa	49	Cabo Verde	Cwa
14	Cabo Verde	Cwa	50	Cabo Verde	Cwa
15	Cabo Verde	Cwa	51	Cabo Verde	Cwa
16	Cabo Verde	Cwa	52	Cabo Verde	Cwa
17	Cabo Verde	Cwa	53	Cabo Verde	Cwa
18	Cabo Verde	Cwa	54	Cabo Verde	Cwa
19	Cabo Verde	Cwa	55	Cabo Verde	Cwa
20	Cabo Verde	Cwa	56	Cabo Verde	Cwa
21	Cabo Verde	Cwa	57	Cabo Verde	Cwa
22	Cabo Verde	Cwa	58	Cabo Verde	Cwa
23	Cabo Verde	Cwa	59	Cabo Verde	Cwa
24	Cabo Verde	Cwa	60	Inconfidentes	Cwa. Cwb
25	Cabo Verde	Cwa	61	Inconfidentes	Cwa. Cwb
26	Cabo Verde	Cwa	62	Inconfidentes	Cwa. Cwb
27	Cabo Verde	Cwa	63	Inconfidentes	Cwa. Cwb
28	Cabo Verde	Cwa	64	Inconfidentes	Cwa. Cwb
29	Cabo Verde	Cwa	65	Inconfidentes	Cwa. Cwb
30	Cabo Verde	Cwa	66	Inconfidentes	Cwa. Cwb
31	Cabo Verde	Cwa	67	Inconfidentes	Cwa. Cwb
32	Cabo Verde	Cwa	68	Inconfidentes	Cwa. Cwb
33	Cabo Verde	Cwa	69	Inconfidentes	Cwa. Cwb
34	Cabo Verde	Cwa	70	Inconfidentes	Cwa. Cwb
35	Cabo Verde	Cwa	71	Inconfidentes	Cwa. Cwb
36	Cabo Verde	Cwa			

[1] Köppen climate classification [50].
